# Supplementary material for: ViraMiner: Deep learning on raw DNA sequences for identifying viral genomes in human samples
Source: PLoS One. 2019 Sep 11;14(9):e0222271. doi: 10.1371/journal.pone.0222271 (PMC6738585; doi:10.1371/journal.pone.0222271)
Supplement: S1 Table — For the most commonly found viral proteins, the table shows how many 300 bp sequences were found in the entire dataset (train+val+test) and same count for only val+test sets. The measures in the last 3 columns are calculated using a combination of validation and test set, validation set was included to increase the number of samples (for more reliable measures). Average score corresponds to the model’s mean output on the given (val+test) sequences. The quartiles show how these scores are distributed. The AUROC is calculated using all test-set non-viral sequences and only the val+test viral sequences corresponding to the given row. For comparison, the baseline values—using all test+val viral sequences, containing genes or not—are 0.390 for average score, [0.016; 0.127; 0.915] for quartiles and 0.923 for AUROC. It can be concluded that the model achieves improved performance on sequences containing these commonly found genes. (PDF) [file pone.0222271.s001.pdf]

**Protein Class**	**Count in all data**	**Count in Test+val set**	**Average score**	**Quartiles [25,50,75]**	**AUROC**
Papillomavirus L1 protein	279	48	0.816	[0.861; 0.985; 0.998]	0.984
Anellovirus orf 1	278	52	0.769	[0.752; 0.976; 0.995]	0.99
Papillomavirus helicase	123	26	0.803	[0.754; 0.990; 0.999]	0.984
Anellovirus ORF2	91	18	0.738	[0.545; 0.975; 0.996]	0.983
Papillomavirus E2 protein, N terminal	83	19	0.574	[0.196; 0.678; 0.943]	0.981
Papillomavirus L2 protein	66	10	0.797	[0.964; 0.983; 0.994]	0.991
Domain of Unknown function (DUF755)	50	10	0.496	[0.023; 0.487; 0.975]	0.964
