# Supplementary material for: ViraMiner: Deep learning on raw DNA sequences for identifying viral genomes in human samples
Source: PLoS One. 2019 Sep 11;14(9):e0222271. doi: 10.1371/journal.pone.0222271 (PMC6738585; doi:10.1371/journal.pone.0222271)
Supplement: S2 Table — The first column shows the viral classes (families) found in the dataset, the second column represents number of viral contigs identified by Blast. The sequences are cut into 300 bp long sequences and the third column counts the numbers after the cut. Cutting longer contigs into smaller pieces means that the resulting 300 bp training sequences represent different parts of the same virus. “Others”, at the last row of table, includes sequences that have by Blast been classified as definitely being viral, but have not been assigned a viral family yet. (PDF) [file pone.0222271.s002.pdf]

Viral class	Number of viruses (before cutting into 300bp)	Number of viruses (after cutting into 300bp)
Anelloviridae	447	1348
Caudovirales	634	1595
Geminiviridae	6	24
Genomoviridae	63	110
Herpesvirales	149	165
Inoviridae	12	15
Circoviridae	4	10
Iridoviridae	5	8
Microviridae	251	252
Mimiviridae	42	67
Papillomaviridae	845	1045
Parvoviridae	37	86
Phycodnaviridae	55	64
Polyomaviridae	14	48
Poxviridae	31	50
Retroviridae	20	24
Others	233	640
